# SIGIRR Mutation in Human Necrotizing Enterocolitis (NEC) Disrupts STAT3-Dependent microRNA Expression in Neonatal Gut

**DOI:** 10.1016/j.jcmgh.2021.09.009

**Published:** 2021-09-23

**Authors:** Wei Yu, Inamul Haque, Aparna Venkatraman, Heather L. Menden, Sherry M. Mabry, Badal C. Roy, Sheng Xia, Jeremy W. Prokop, Shahid Umar, Aron M. Geurts, Venkatesh Sampath

**Affiliations:** 1 Division of Neonatology, Department of Pediatrics, Children's Mercy Hospital, Kansas City, Missouri; 2 Department of Surgery, University of Kansas Medical Center, Kansas City, Kansas; 3 Department of Pediatrics and Human Development, Michigan State University, Grand Rapids, Michigan; 4 Department of Physiology, Medical College of Wisconsin, Milwaukee, Wisconsin; 5 Genomics Sciences and Precision Medicine Center, Medical College of Wisconsin, Milwaukee, Wisconsin; 6 Center of Systems Molecular Medicine, Medical College of Wisconsin, Milwaukee, Wisconsin

**Keywords:** SIGIRR, microRNA, STAT3, Intestinal Inflammation, cDNA, complementary DNA, ChIP, chromatin immunoprecipitation, DOL, day of life, FBS, fetal bovine serum, HIEC, human intestinal epithelial cell, IEC, intestinal epithelial cell, IL, interleukin, IL1R, interleukin-1 receptor, IRAK1, interleukin-1–related–associated kinase 1, miRNA, microRNA, MYD88, myeloid differentiation primary response 88, NEC, necrotizing enterocolitis, NF-κB, nuclear factor-κB, PBS, phosphate-buffered saline, PCR, polymerase chain reaction, shRNA, short hairpin RNA, siRNA, small interfering RNA, SIGIRR, single immunoglobulin interleukin-1–related receptor, STAT3, signal transducer and activator of transcription 3, TIR, Toll/interleukin-1 receptor, TLR, Toll-like receptor, CRISPR/Cas9, (clustered regularly interspaced short palindromic repeats), ACTB, actin beta

## Abstract

**Background & Aims:**

Single immunoglobulin interleukin-1–related receptor (SIGIRR) is a major inhibitor of Toll-like receptor signaling. Our laboratory identified a novel SIGIRR stop mutation (p.Y168X) in an infant who died of severe necrotizing enterocolitis (NEC). Herein, we investigated the mechanisms by which SIGIRR mutations induce Toll-like receptor hyper-responsiveness in the neonatal gut, disrupting postnatal intestinal adaptation.

**Methods:**

Clustered regularly interspaced short palindromic repeats (CRISPR)/Cas9 was used to generate transgenic mice encoding the SIGIRR p.Y168X mutation. Ileal lysates, mouse intestinal epithelial cell (IEC) lysates, and intestinal sections were used to assess inflammation, signal transducer and activator of transcription 3 (STAT3) phosphorylation, microRNA (miRNA), and interleukin-1–related–associated kinase 1 (IRAK1) expression. Western blot, quantitative reverse-transcription polymerase chain reaction(qRT-PCR), and luciferase assays were performed to investigate SIGIRR–STAT3 signaling in human intestinal epithelial cells (HIEC) expressing wild-type or SIGIRR (p.Y168X) plasmids.

**Results:**

*Sigirr*^*Tg*^ mice showed increased intestinal inflammation and nuclear factor-κB activation concomitant with decreased IEC expression of miR-146a and miR-155. Mechanistic studies in HIECs showed that although SIGIRR induced STAT3-mediated expression of miR-146a and miR-155, the p.Y168X mutation disrupted SIGIRR-mediated STAT3-dependent miRNA expression. Chromatin immunoprecipitation and luciferase assays showed that SIGIRR activation of STAT3-induced miRNA expression is dependent on IRAK1. Both in HIECs and in the mouse intestine, decreased expression of miR-146a observed with the p.Y168X mutation increased expression of IRAK1, a protein whose down-regulation is important for postnatal gut adaptation.

**Conclusions:**

Our results uncover a novel pathway (SIGIRR–STAT3–miRNA–IRAK1 repression) by which SIGIRR regulates postnatal intestine adaptation, which is disrupted by a SIGIRR mutation identified in human NEC. These data provide new insights into how human genetic mutations in SIGIRR identified in NEC result in loss of postnatal intestinal immune tolerance.

## Graphical abstract

**Figure:**
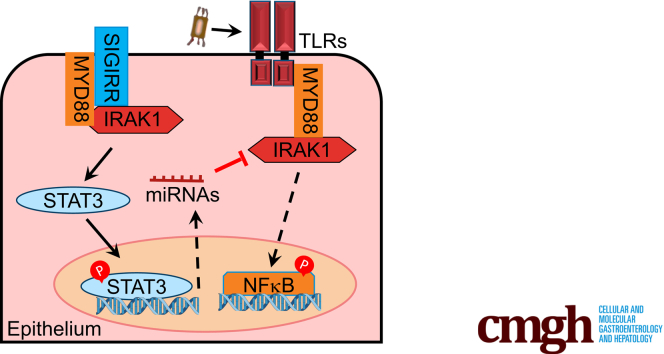

**Summary**

How host genetics regulates neonatal intestinal adaptation is unclear. Investigating a single immunoglobulin interleukin-1–related receptor (SIGIRR) mutation identified in an infant with necrotizing enterocolitis reveals that signal transducer and activator of transcription 3 (STAT3)–microRNA–mediated repression of interleukin-1–related associated kinase 1(IRAK1) protein is lost with SIGIRR mutation. This results in deviant Toll-like receptor signaling and loss of postnatal intestinal adaptation.

Preterm infants are at increased risk of necrotizing enterocolitis (NEC), characterized pathologically by intestinal necrosis and inflammation. NEC develops in 5%–14% of preterm infants born before 30 weeks' gestation and has a mortality rate of 20%–35%.^1^ Although the pathogenesis of NEC remains unclear, genetic, nutritional, and environmental risk factors that favor deviant interactions between the intestinal mucosa and gut microbiota portend NEC vulnerability.^1^, ^2^, ^3^, ^4^ Animal models suggest that aberrant activation of intestinal Toll-like receptor 4 (TLR4), a sensor of lipopolysaccharide derived from gram-negative bacteria is a central event in NEC pathogenesis, and *TLR4-/-* mice are protected against experimental NEC.^5^^,^^6^ Studies on human intestinal tissues derived from preterm infants with NEC also have suggested that genes that mediate TLR signaling such as TLR4, myeloid differentiation primary response 88 (MYD88), and downstream cytokines are increased in NEC, while negative regulators of TLR signaling such as single immunoglobulin interleukin-1–related receptor (SIGIRR) and A20 have decreased expression in NEC.^7^ Whether a native state of TLR4 hyper-responsiveness that favors intolerance to colonizing bacteria in the developing intestine exists, and the factors that prime TLR hypersensitivity, remain unknown.^8^^,^^9^

After birth, the neonatal intestinal mucosa is exposed to commensal and pathogenic microbial organisms recognized by innate immune receptors, such as TLRs. TLRs contribute to antimicrobial host defense and intestinal homeostasis,^10^ but aberrant activation of TLR signaling, notably TLR4, has been implicated in mucosal injury and inflammation underlying NEC and other diseases.^5^^,^^8^^,^^9^ TLR-related signaling in intestinal epithelial cells (IECs) must be tightly regulated to protect the neonatal gut from TLR hypersensitivity and inflammation induced by gut microbiota. Down-regulation and apical to basal localization of TLR4 and postnatal decrease in the expression of the key TLR canonical signaling kinase, interleukin-1–receptor–associated kinases 1 (IRAK1), are some mechanisms facilitating postnatal intestinal tolerance. Enhanced expression of negative regulators of TLR4 signaling also promote intestinal mucosal tolerance to bacteria.^7^^,^^11^^,^^12^ SIGIRR, a major negative regulator of TLR signaling, is an orphan receptor composed of an extracellular domain, transmembrane domain, and intracellular Toll/interleukin-1 receptor (TIR) domain. SIGIRR inhibits TLR signaling by competitively binding to MYD88, the major TLR adapter, through its TIR domain, and inhibiting TLR–MYD88 signal transduction. SIGIRR has been implicated in NEC pathogenesis. Our laboratory identified multiple *SIGIRR* mutations in preterm NEC patients, and we have posited that loss of functional SIGIRR variants may increase the vulnerability of preterm infants to TLR-mediated inflammation and NEC.^3^^,^^13^ Whether *SIGIRR* variants alter postnatal intestinal tolerance and the mechanisms by which SIGIRR genetic variants contribute to postnatal intestinal adaptation remain unanswered. In this study, we developed a novel *Sigirr* mutant mouse that recapitulates a truncating mutation (p.Y168X) we previously identified in an infant with NEC to investigate this question.^13^

TLR signaling also can be inhibited at the post-transcriptional level by microRNAs (miRNA), small noncoding RNA of approximately 2 nucleotides. Anti-inflammatory miRNAs, such as miR-146a and miR-155, target TLR pathway components required for signal transduction, and are expressed abundantly in the intestinal epithelium.^14^^,^^15^ Sustained intestinal expression of miR-146a in the intestinal epithelium is essential for establishment of innate immune tolerance to colonizing bacteria.^16^ The mechanisms that regulate miR-146a expression in neonatal intestine are incompletely understood. Understanding regulation of anti-inflammatory microRNA expression is directly relevant to NEC pathogenesis because imbalances in expression of proinflammatory vs anti-inflammatory miRNAs have been reported in intestinal tissues obtained from preterm infants with NEC.^17^^,^^18^ Based on knowledge described earlier, we hypothesized that SIGIRR may regulate TLR sensitivity and IRAK1 expression in neonatal intestinal epithelium through microRNA. Herein, we show that SIGIRR regulates miR-146a expression through IRAK1-mediated signal transducer and activator of transcription 3 (STAT3)-dependent transcriptional activation both in cultured primary human intestinal epithelial cells and in neonatal small intestine.

## Results

### SIGIRR Regulates Expression of microRNAs in Primary Human IECs

**Figure 1:**
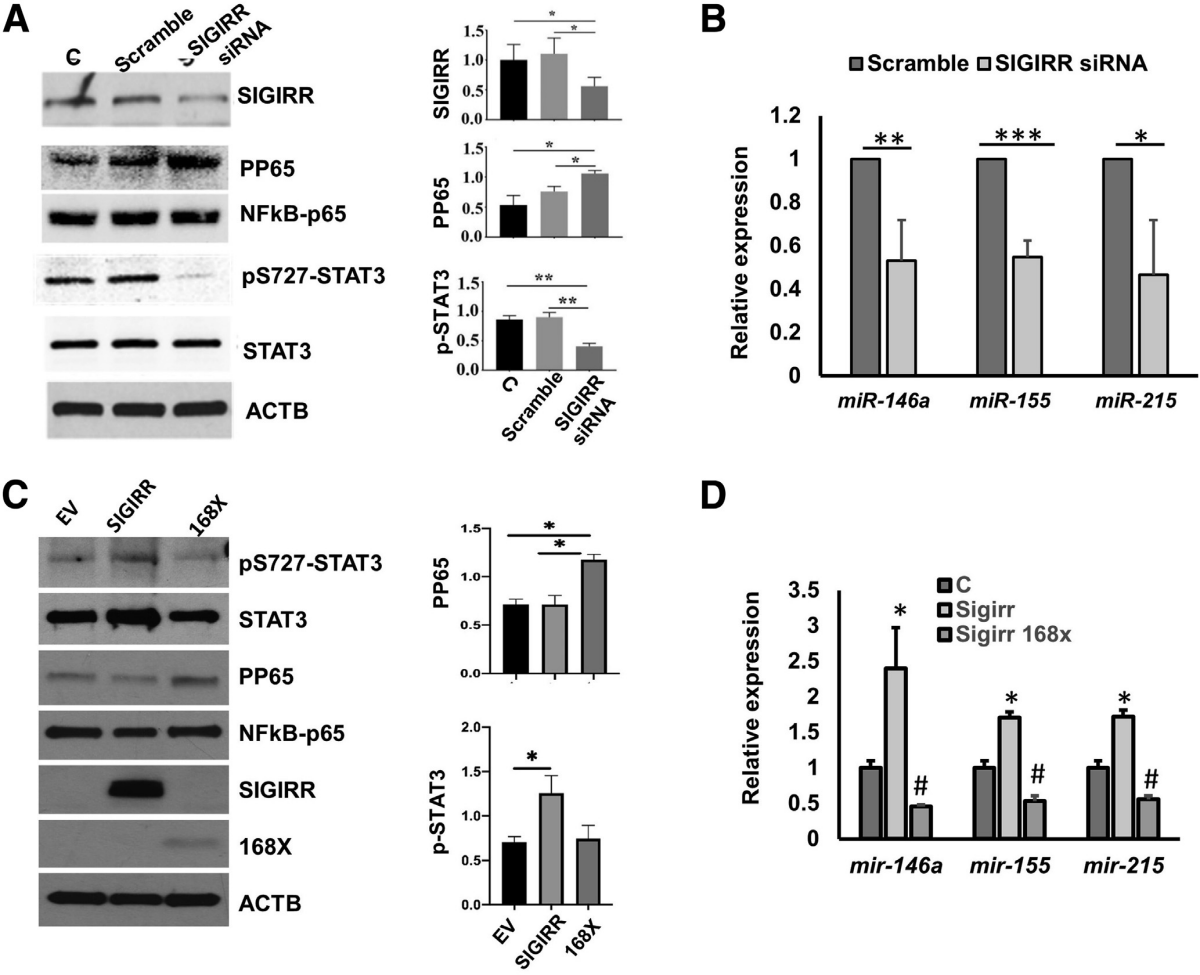
**SIGIRR is required for anti-inflammatory microRNA expression in HIECs.** (*A*) HIEC lysates of nontransfected(C, control), transfected with Scramble siRNA and SIGIRR siRNA were analyzed by Western blot using the indicated antibodies, with densitometry quantification shown graphically. (*B*) microRNA expression was quantified by real-time PCR in HIECs transfected with scramble or SIGIRR siRNA. (*C*) HIECs were transfected with pcDNA3–empty vector (EV), pcDNA3–SIGIRR, and pcDNA3–SIGIRR p168X mutant, and the cell lysates were analyzed by Western blot using the indicated antibodies. The densitometry quantification is shown graphically. (*D*) microRNA expression was quantified by real-time PCR in HIECs with indicated transfection. ∗*P* < .05, ^#^*P* < .05, ∗∗*P* < .01, ∗∗∗*P* < .005. ACTB, actin beta.

To determine whether SIGIRR regulates the expression of anti-inflammatory microRNAs, an RNA interference–based approach was used. Three days after transfection of human intestinal epithelial cells (HIECs) with SIGIRR-targeting small interfering RNA (siRNA) (SIGIRR siRNA), SIGIRR abundance was reduced by approximately 60% compared with the control (untransfected) group (Figure 1*A*). HIECs treated with SIGIRR siRNA showed 50% decreased expression of anti-inflammatory miR-146a and miR-155 than did control siRNA-transfected cells, as detected by quantitative polymerase chain reaction (PCR) (Figure 1*B*). Because mir-215 has been shown to protect against lipopolysaccharide-induced inflammation in myoblast cells, and is decreased in human NEC intestine,^17^^,^^19^ mir-215 expression level also was examined. Similar to the other 2 microRNAs, mir-215 expression decreased in HIECs with SIGIRR knockdown (Figure 1*B*). We next examined whether SIGIRR mediated microRNA expression through nuclear factor-κB (NF-κB). SIGIRR-silencing induced p65 (RELA proto-oncogene, NF-κB subunit(RELA)) phosphorylation as anticipated in parallel with decreased miRNA expression (Figure 1*A* and *B*), excluding its role in positive regulation of miRNAs. STAT3 is a well-studied transcription factor antagonizing proinflammatory signals during innate immune response,^20^ and is known to regulate expression of miRNAs in human hepatocellular carcinoma cells and T-helper 17 cells,^21^^,^^22^ so expression of STAT3 and its phosphorylated form were examined. We noted that serine phosphorylation of STAT3 at residue 727 (STAT3^Ser727^), a major regulation site for STAT3 transcriptional activation, was reduced in SIGIRR-knockdown HIECs, while the total STAT3 level remain unchanged (Figure 1*A*).

To assess direct relevance of SIGIRR mutations identified in NEC infants on SIGIRR-dependent STAT3 phosphorylation and miRNA expression, we focused on the SIGIRR stop mutation that truncates the intracellular TIR domain at residue Y168^13^ (Figure 3*F*). STAT3^Ser727^ phosphorylation and anti-inflammatory microRNAs were induced in HIECs transfected with wild-type SIGIRR compared with HIECs expressing empty plasmid (Figure 1*C*). SIGIRR p.Y168X mutant failed to promote microRNA expression (Figure 1*D*), but repressed their expression instead, indicating that the intracellular domain of SIGIRR may be required for signal transduction to activate STAT3^Ser727^ phosphorylation and anti-inflammatory microRNAs expression. The 2.5- to 3.0-fold increase in miRNA expression after SIGIRR transfection was relatively modest, but was consistent. This potentially arose from decreased efficiency of transient transfections, and variation in timing of the peak change. These data indicate that SIGIRR regulates baseline expression of anti-inflammatory miRNA in association with STAT3 phosphorylation, and human SIGIRR mutations that truncate the TIR domain abolish SIGIRR-dependent miRNA expression.

### STAT3 Directly Binds to microRNA Promoters and Activates Expression in a SIGIRR- Dependent Manner

**Figure 2:**
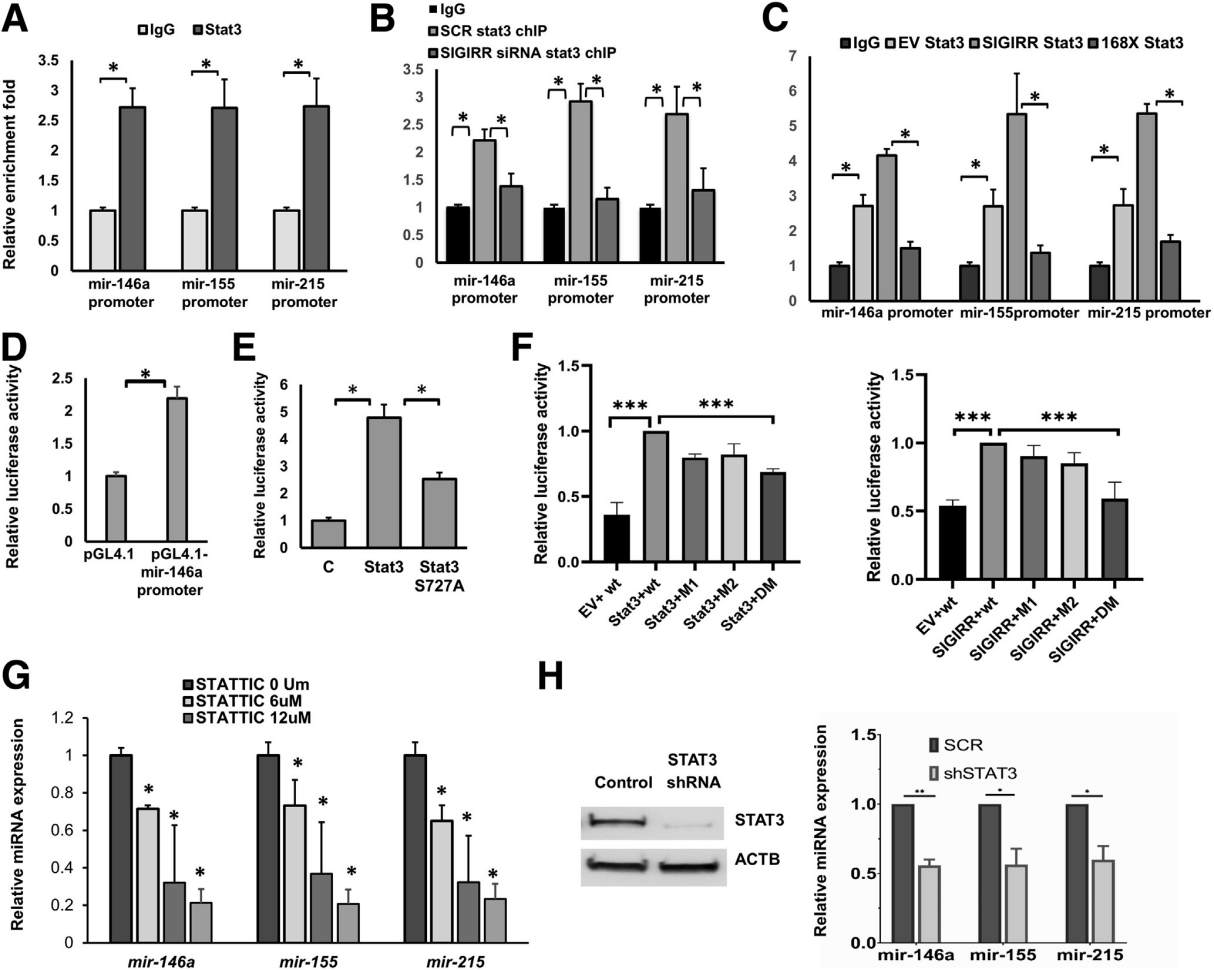
**STAT3 activates microRNA expression in a SIGIRR-dependent manner.** (*A*) Quantitative-ChIP assay was performed using anti-STAT3 antibody to analyze the recruitment of STAT3 on promoters of miR-146a, miR-155, and mir-215 in HIECs. Values were quantified against IgG controls. (*B* and *C*) ChIP quantitative PCR values of STAT3 recruitment on microRNA promoters in HIECs with indicated transfection. (*D*) Promoter reporter activity assay was performed in HEK293T cells transfected with luciferase reporter plasmid pGL4.1 empty vector or pGL4.1 containing human miR-146a promoter, and thymidine kinase promoter–Renilla luciferase reporter plasmid (pRL-TK), serving as a internal transfection control. Luciferase assay was performed with cell lysates using the Dual-Luciferase Reporter Assay System(Promega, Madison, WI). (*E*) Promoter reporter activity assays were performed in HEK293T cells co-transfected with pGL4.1–miR-146a promoter and indicated STAT3 plasmids. (*F*) Promoter reporter activity assays were performed in HEK293T cells using PGL4.1–miR-146a promoter with nucleotide mutations at STAT3 binding sites. HEK293T were co-transfected with wild-type miR-146a promoter luciferase reporter or mutant reporter. The relative luciferase activities obtained from 5 to 6 independent experiments are shown as means ± SD. (*G*) microRNA expression was quantified by real-time PCR in HIECs treated with STAT3 inhibitor, STATTIC, for 24 hours at the indicated concentration. (*H*) microRNA expression was quantified by real-time PCR in HIECs transduced with control shRNA or Stat3 shRNA. ∗*P* < .05, ∗∗∗*P* < .005. ACTB, actin beta; DM, mutations at STAT3 binding site 1 and site 2; EV, pcDNA3–empty vector; M1, mutations at STAT3 binding site 1; SCR, scrambled; SIGIRR, pcDNA3–SIGIRR; 168X, pcDNA3–SIGIRR P168X.

The 2-kb promoter of miR-146a, miR-155, and mir-215 was predicted to possess multiple Stat3 binding sites by JASPAR (http://jaspar.genereg.net). The binding of STAT3 on promoters of microRNA was evident by chromatin immunoprecipitation (ChIP) assay using Stat3-specific antibody followed by qPCR with primers targeting microRNA promoter regions predicted to have the highest binding score (Figure 2*A*). We next examined the influence of SIGIRR on the binding affinity of STAT3 microRNAs in HIECs transfected with SIGIRR siRNA. As shown, STAT3 binding to microRNA promoter was abolished in SIGIRR siRNA-transfected HIECs compared with control siRNA-transfected HIECs (Figure 2*B*). Expression of SIGIRR p.Y168X mutant also decreased the binding affinity of Stat3 to the microRNA promoter (Figure 2*C*).

To determine whether STAT3 functions as a transcriptional activator for microRNAs, we generated a luciferase reporter by inserting the 2-kb miR-146a promoter before the luciferase coding fragment. Co-transfection with a wild-type STAT3 plasmid strongly activated miR-146a promoter activity. Strikingly, the phosphorylation defective S727A mutant of STAT3 only displayed approximately 50% transcriptional activation compared with wild-type STAT3 (Figure 2*E*). To define their role in miRNA promoter activation, STAT3 binding sites at miR-146a promoter were mutated by site-directed PCR mutagenesis. Because of multiple potential STAT3 binding sites, we chose to mutate the 2 sites showing the highest binding affinity scores. Reporter activation by wild-type STAT3 was attenuated nonsignificantly when individual STAT3 binding sites at the miR-146a promoter were deleted, and significantly when both sites were deleted (Figure 2*F*). Importantly, SIGIRR was unable to activate miR-146a promoter activity when both STAT3 promoter binding sites on miR-146a were deleted (Figure 2*F*). STAT3 regulation of miRNA expression was tested in HIECs by blocking STAT3 activation using a small-molecule inhibitor named Stattic, which selectively inhibits activation, dimerization, and nuclear translocation of STAT3.^23^ MicroRNA expression was strikingly decreased in a dose-dependent manner after 16 hours of STAT3 inhibitor treatment (Figure 2*G*). Alternatively, knocking-down STAT3 using short hairpin RNA (shRNA)-based RNA interference reduced miRNA expression in HIECs (Figure 2*H* and *I*). Taken together, these results indicate that SIGIRR regulates STAT3-dependent anti-inflammatory microRNA promoter activity and expression at baseline without exogenous ligand stimulation.

### IRAK1-Dependent Phosphorylation of Stat3 Is Required for miRNA Expression in HIECs

**Figure 3:**
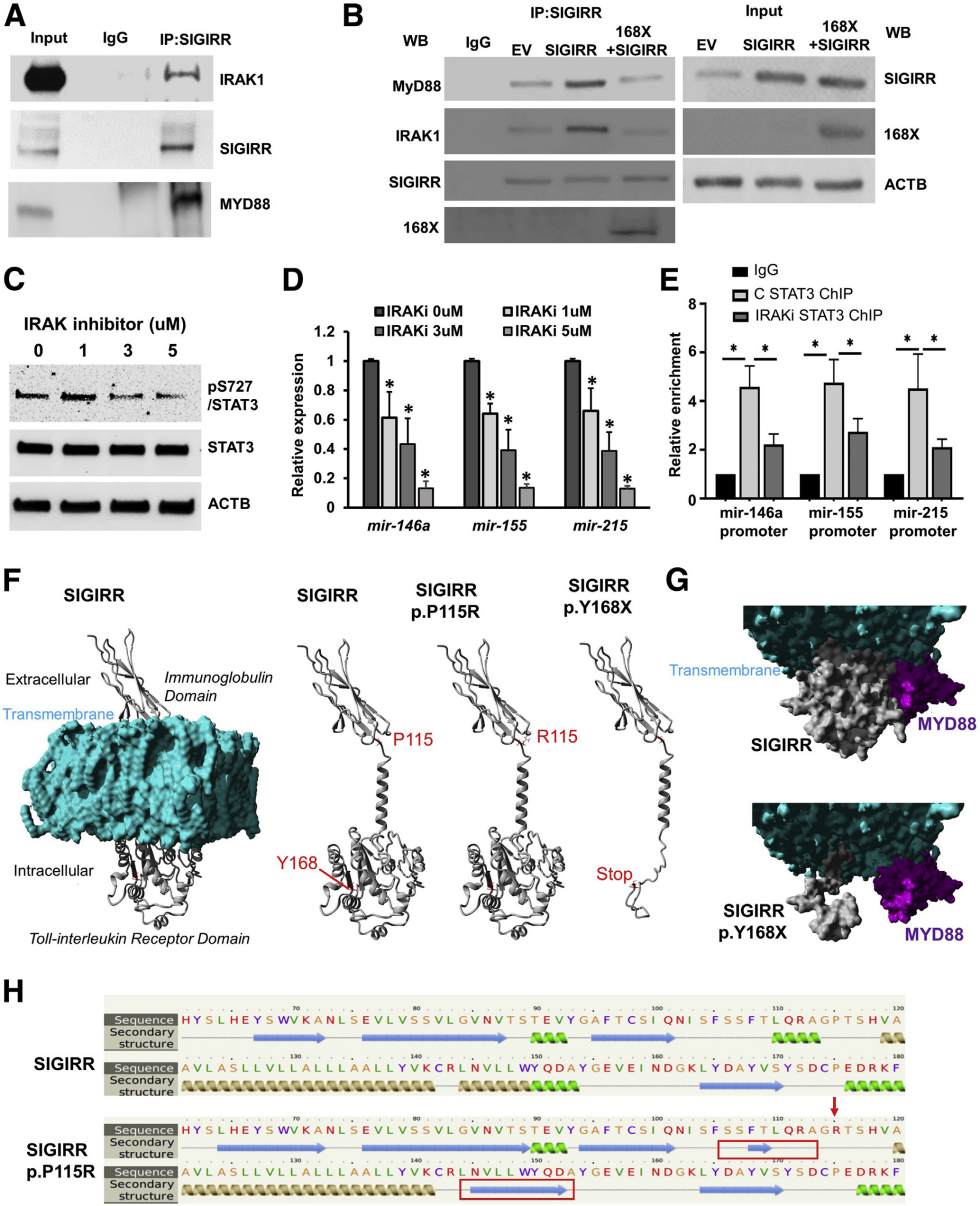
**STAT3 phosphorylated by IRAK1 regulates microRNA expression.** (*A*) SIGIRR interacts specifically with IRAK1 and MYD88, but not TLRs in HIECs at baseline. HIEC lysates were immunoprecipitated by SIGIRR antibody, then immunoprecipitated complexes were immunoblotted with the indicated antibodies. (*B*) HIECs were transfected with pcDNA3–empty vector (EV), pcDNA3–SIGIRR (SIGIRR), and pcDNA3–SIGIRR plus pcDNA3-SIGIRR p168X mutant, immunoprecipitation (IP) was performed using SIGIRR antibody and analyzed by Western blot with the indicated antibodies. (*C* and *D*) HIECs were treated with the IRAK1/4 inhibitor at the escalated concentrations for 24 hours, then were lysed for Western blot (WB) and real-time PCR analysis of microRNA expression. (*E*) ChIP quantitative PCR values of STAT3 recruitment on microRNA promoters in HIECs treated with IRAK1/4 inhibitor for 16 hours. (*F–H*) SIGIRR protein models for study. (*F*) The far-left structure shows the full mature SIGIRR protein structure (amino acids 1–410, Q6IA17; UniProt) within a lipid membrane (cyan), with labeled domains and portions of the cell. The next model shows the full protein without the membrane and labeling in red amino acid P115 and Y168. The next 2 models show SIGIRR p.P115R and p.Y168X. (*G*) Molecular surface plot of the intracellular Toll-interleukin receptor domain (gray) for the full SIGIRR or SIGIRR p.Y168X interacting with MYD88 (magenta). (*H*) Predicted effect of the SIGIRR p.P115R mutant on the first α helix of the intracellular TIR domain. The predicted change of protein structure in SIGIRR p.P115R mutant is marked by red boxes. ∗*P* < .05. ACTB, actin beta; C , control.

To examine how SIGIRR mediates STAT3 phosphorylation, we targeted IRAK1, positing that the TIR domain of SIGIRR can interact with MYD88 and IRAK1 to mediate signal transduction. Co-immunoprecipitation studies showed that SIGIRR co-immunoprecipitated with IRAK1 and MYD88 while normal IgG failed to yield either protein, suggesting that SIGIRR forms a complex with IRAK1 and MYD88 independent of exogenous TLR ligand stimulation (Figure 3*A* and *G*). Further corroboration was provided with studies showing that overexpression of SIGIRR wild-type protein enhanced binding with IRAK1 and MYD88. The SIGIRR mutation identified in NEC, which lacks the TIR domain, interfered with wild-type SIGIRR binding to IRAK1 and MYD88 (Figure 3*B*), indicating it may function as a dominant-negative mutant. IRAK1 dependency of STAT3 Ser727 phosphorylation was examined using a selective inhibitor of IRAK1/4 kinase activity. STAT3^Ser727^ phosphorylation was inhibited 16 hours after treatment with the IRAK1/4 inhibitor in a dose-dependent manner (Figure 3*C*). In parallel with decreased STAT3^Ser727^ phosphorylation, anti-inflammatory microRNA expression decreased with escalating doses of IRAK1 inhibitor (Figure 3*D*). ChIP assays showed that IRAK1 inhibitor treatment prevented STAT3 binding to the microRNA promoter, which resulted in less transcriptional activation of microRNAs (Figure 3*E*). Taken together, our results suggest that SIGIRR binds to IRAK1, and directs IRAK1-dependent, STAT3-mediated transcriptional activation of miRNA expression.

### SIGIRR-Mediated Inhibition of TLR4 Signaling Is Dependent on Stat3–miRNA Activation

**Figure 4:**
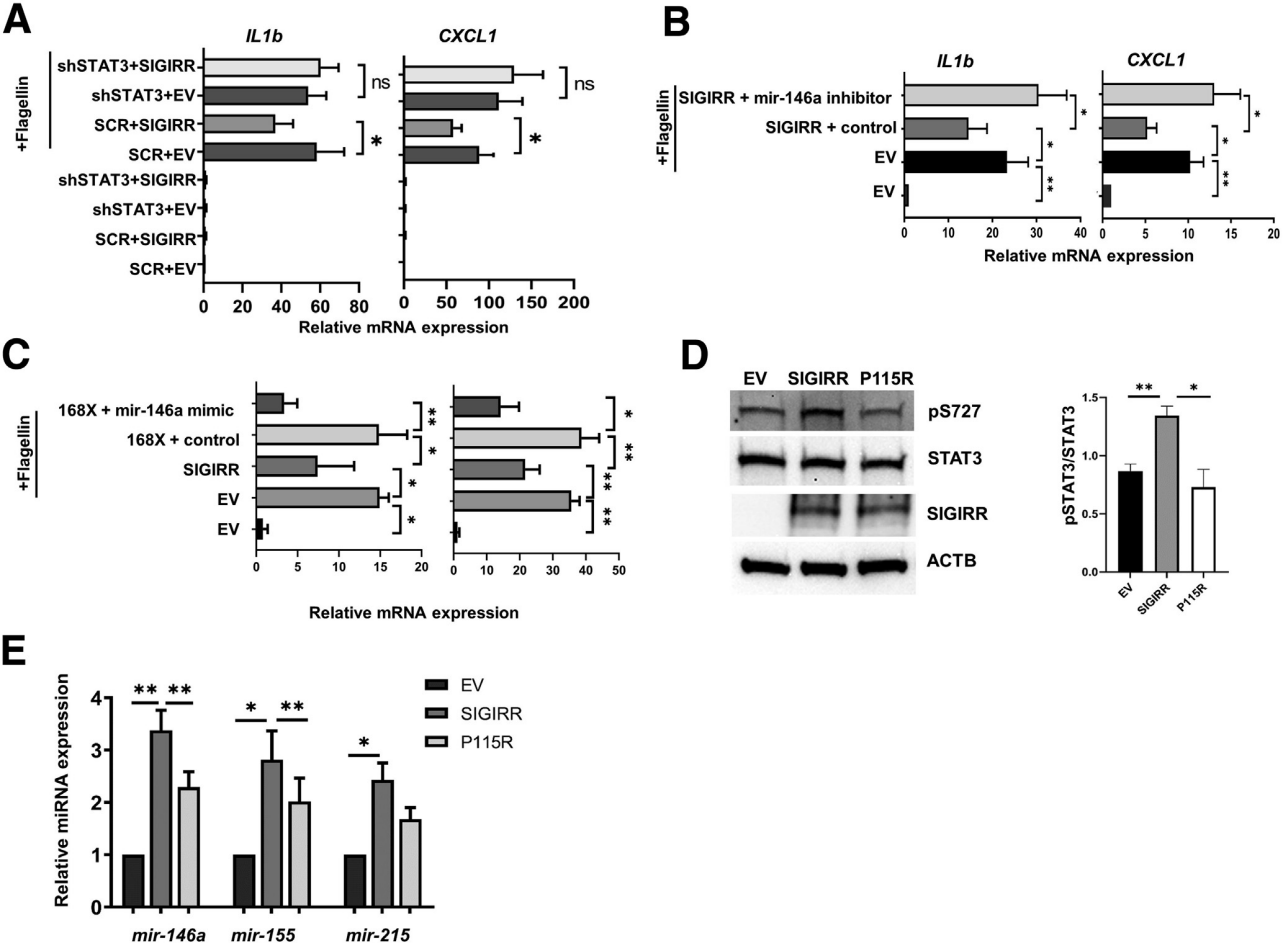
**SIGIRR suppresses TLR-induced inflammation partially via anti-inflammatory microRNAs.** (*A*) SIGIRR inhibits flagellin-induced proinflammatory cytokine expression through STAT3. HIECs stably expressing lentiviral Stat3 shRNA or scramble shRNA (SCR) were transfected with pcDNA3–empty vector (EV) or pcDNA3-SIGIRR for 48 hours, then treated with flagellin at 50 ng/mL for 8 hours. Proinflammatory cytokines and microRNA expression were quantified by real-time PCR. (*B*) miR-146a inhibitor restores IL1b and C-X-C motif chemokine ligand 1 (CXCL1) expression suppressed by SIGIRR. HIECs were treated with flagellin for 8 hours after 48 hours of transfection of pcDNA3-SIGIRR and miR-146a inhibitor or scramble control. IL1b and CXCL1 expression were analyzed by real-time PCR. (*C*) HIECs were transfected with plasmids and miR-146a mimic or scramble control as indicated for 48 hours, then treated with flagellin for 6 hours at 100 ng/μL. IL1b and CXCL1 expression were analyzed by real-time PCR. (*D*) HIECs were transfected with pcDNA3–EV, pcDNA3–SIGIRR, and pcDNA3–SIGIRR p.P115R mutant, and cell lysates were analyzed by Western blot using the indicated antibodies. Densitometry quantification is shown graphically. (*E*) microRNA expression was quantified by real-time PCR in HIECs with indicated transfection. ∗*P* < .05, ∗∗*P* < .01, ACTB, actin beta.

SIGIRR is known to mediate inhibition of TLR signaling by disrupting TIR–TIR interactions between TLR receptors and adapter MYD88. We probed the functional relevance of the SIGIRR–STAT3–miRNA axis to suppression of proinflammatory TLR signaling in HIECs. To investigate this, we used a lentiviral shRNA knock strategy to generate HIECs (HIEC^***Stat3-***^) with more than 60% stable knockdown of STAT3 (Figure 2*H*). Plasmid-mediated expression of SIGIRR in HIEC^*Stat3-*^ did not suppress flagellin (TLR5 agonist)-induced interleukin (IL)1β and C-X-C motif chemokine ligand 1 expression at 16 hours to the extent observed in HIECs (HIEC^*Stat3+*^) transduced with scrambled shRNA lentivirus-vector (Figure 4*A*). To further prove miRNAs are involved in SIGIRR-mediated suppression of TLR5-induced inflammation, we used miR-146a inhibitor to block miR-146a function in HIECs and found that SIGIRR was not able to suppress flagellin-induced inflammatory cytokine expression (Figure 4*B*). Besides blocking SIGIRR function using miR-146a inhibitor, we also used miR-146a mimic to rescue function of the p.Y168X variant in TLR signaling suppression. Strikingly, we observed that the miR-146a mimic rescued SIGIRR function to suppress flagellin-induced cytokine expression in HIECs (Figure 4*C*).

Besides the SIGIRR p.Y168X stop mutant, we also identified other genetic variants associated with human NEC previously.^13^ The SIGIRR p.P115R variant was predicted (The Phyre2 web portal , http://www.sbg.bio.ic.ac.uk/phyre2/html/page.cgi?id=index ) to alter the structure of the first α-helix of the TIR intracellular domain (Figure 3*F* and *H*). We therefore evaluated the effect of the p.P115R SIGIRR variant identified in NEC on regulating anti-inflammatory miRNA expression. Compared with wild-type SIGIRR, the p.P115R variant had reduced ability to induce STAT3 (Ser727) phosphorylation or microRNA expression, and did not suppress flagellin-induced inflammation (Figure 4*D* and *E*). These data suggest that SIGIRR human mutations that disrupt function of the intracellular TIR domain have decreased ability to induce STAT3-dependent miRNA expression.

### *SIGIRR* Mutant Mice Encoding a Truncated TIR Domain (p.P174X) Show Spontaneous Neonatal Intestinal Inflammation With Decreased microRNA Expression

**Figure 5:**
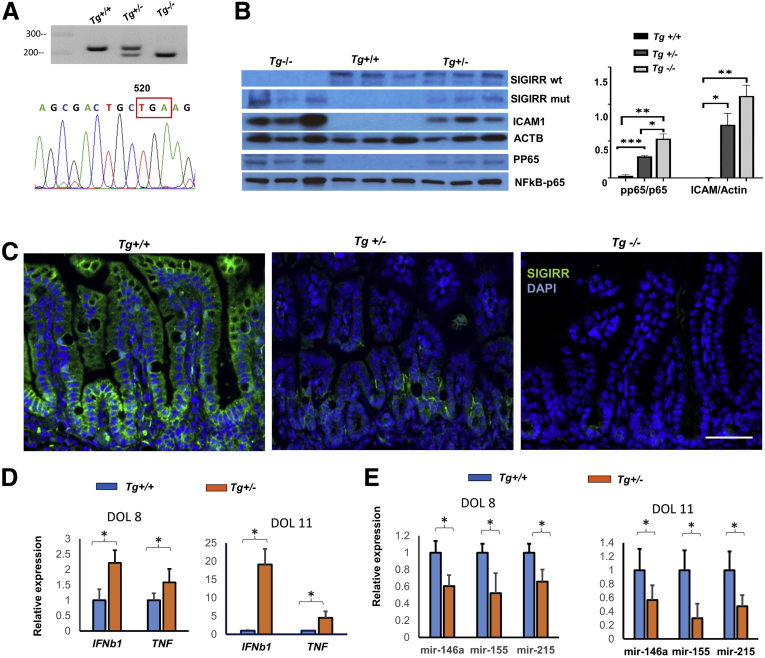
**Spontaneous inflammation with low-level microRNA expression in small intestine of neonatal *Sigirr*^*Tg*^ mutant mice.** (*A*) Genotyping of *Sigrr*^*Tg*^ mice and sequencing of *Sigirr* cDNA from mutant mice was performed to confirm the deletion of TIR domain. *Tg*+/+ represents the wild type, *Tg*+/- and *Tg*-/- represent mice heterozygous or homozygous for the *Sigirr* p.P174X mutant, respectively. The premature stop codon in SIGIRR gene is marked by red box. (*B*) Whole lysate protein obtained from terminal ileum of littermate DOL-11 *Sigrr*^*Tg*^ mutant mice was immunoblotted for indicated antibodies, with densitometry quantification shown graphically. (*C*) Immunofluorescent confocal images of DOL-11 mouse terminal ileum stained with SIGIRR antibody. (*D*) Proinflammatory cytokines were quantified by real-time PCR in terminal ileum of DOL-8 and DOL-11 *Sigirr*^*Tg*^ mice. (*E*) microRNA expression in terminal ileum of DOL-8 and DOL-11 *Sigirr*^*Tg*^ mice was quantified by real-time PCR. ∗*P* < .05, ∗∗*P* < .01, ∗∗∗*P* < .005. ACTB, actin beta; DAPI, 4′,6-diamidino-2-phenylindole; ICAM1, intercellular adhesion molecule 1; TNF, tumor necrosis factor.

**Figure 6:**
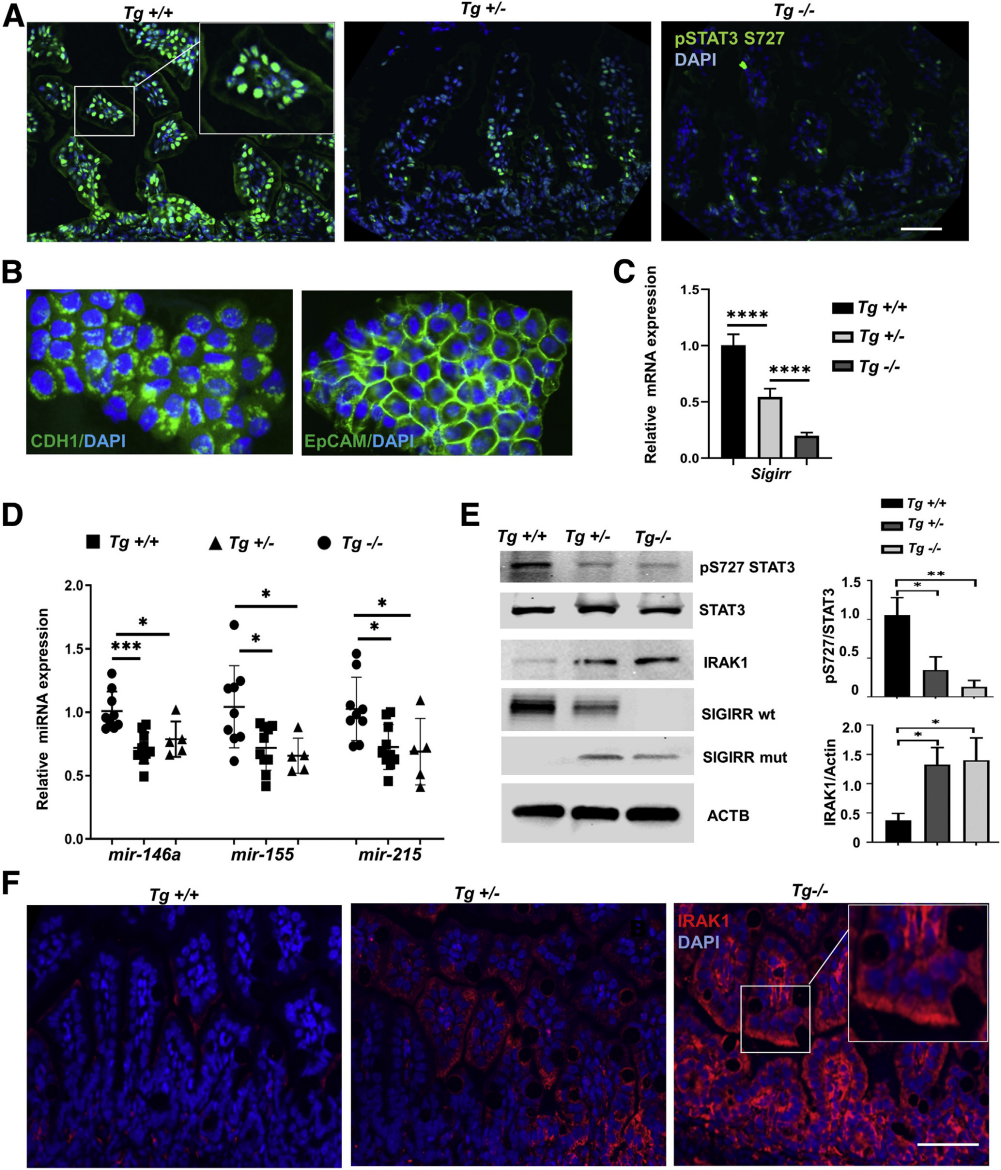
**Decreased intestinal expression of microRNAs and increased TLR signaling molecules in *Sigirr*^*Tg*^ mice.** (*A*) Immunofluorescent confocal images of DOL-11 mouse terminal ileum stained with phospho-STAT3 (Ser727) antibody. (*B*) Immunofluorescent confocal images of isolated IECs from DOL-11 mouse terminal ileum stained with epithelial cell markers. (*C*) *Sigirr* mRNA expression in isolated IECs. (*D*) microRNA expression in IECs isolated from terminal ileum of DOL-12 *Sigirr*^*Tg*^ mice was quantified by real-time PCR. (*E*) Mouse IEC lysates from littermate *Sigirr*^*Tg*^ mice were analyzed by immunoblotting with indicated antibodies, with densitometry quantification shown graphically. (*F*) Immunofluorescent confocal images of DOL-11 mouse terminal ileum stained with IRAK1 antibody. ∗*P* < .05, ∗∗*P* < .01, ∗∗∗*P* < .005, ∗∗∗∗*P*< .001. ACTB, actin beta; DAPI, 4′,6-diamidino-2-phenylindole;

Loss of the SIGIRR TIR domain ensuing from the p.Y168X mutation identified in human NEC resulted in loss of STAT3-mediated microRNA expression and exaggerated TLR5 responsiveness in HIECs. To investigate the in vivo ramifications of this finding we used clustered regularly interspaced short palindromic repeats (CRISPR)-Cas9 technology to generate a novel transgenic mouse encoding a premature stop codon at the *Sigirr* locus to mimic the human truncating mutation p.Y168X (Figure 5*A*). The mutation in mice (*Sigirr*^*Tg*^) genocopies the human mutation by truncating the mouse intracellular TIR domain at amino acid residue 174 (Figure 5*A*). The truncating mutation resulted in a protein that was 18 kilodaltons, compared with full-length SIGIRR, which was 54 kilodaltons (Figure 5*B*). Immunofluorescence studies showed that SIGIRR was expressed along the crypt–villus axis, both at the apical and basal aspects of mouse IECs. Interestingly, the SIGIRR mutant resulted in decreased SIGIRR protein expression in both crypt and villi (Figure 5*C*), which also was confirmed by quantitative RNA analysis of the SIGIRR transcript in IEC lysates (Figure 6*C*). This suggests that truncation results in nonsense-mediated decay, resulting in decreased levels of truncated SIGIRR RNA and protein. Strikingly, we noted a dose-dependent increase in spontaneous intestinal inflammation in the terminal ileum, even with heterozygous neonatal pups showing increased expression of cytokines, such as *Ifnb1* and *Tnfα*, compared with littermate wild types (Figure 5*D*). Intercellular adhesion molecule expression as well as markers of canonical TLR pathway signaling such as p65 (RELA) phosphorylation were induced in the terminal ileum of heterozygous and homozygous *Sigirr*^*Tg*^ mice on day of life (DOL)-8 (Figure 5*B*). These data suggest that in mice encoding a SIGIRR TIR domain-truncating mutation, there is TLR hypersensitivity with increased ileal intestinal cytokine expression and TLR canonical pathway activation.

### *Sigirr*^*Tg*^ Mice Have Decreased Intestinal Expression of microRNAs and Phospho-STAT3 but Have Increased Expression of IRAK1

We next investigated the relationship between SIGIRR and microRNA expression in the developing intestine. Loss of SIGIRR expression was accompanied by a concomitant dose-dependent decrease in phosphorylated STAT3 Ser727 expression (Figure 6*A*). IECs were isolated from neonatal terminal small intestine and epithelial identity confirmed by immunofluorescence staining for epithelial cell adhesion molecule (EpCAM) and E-cadherin (Figure 6*B*). Heterozygous and homozygous *Sigirr*^*Tg*^ IECs showed reduced expression of all miRNAs on DOL-11 (Figure 6*D*). miRNA expression also decreased in lysates obtained from the terminal ileum of *Sigirr*^*Tg*^ mice on DOL-8 and DOL-11 (Figure 5*E*). These data show that in *Sigirr*^*Tg*^ mice, decreased expression of anti-inflammatory miR-146a and miR-155 is associated with spontaneous TLR pathway activation. Other investigators have shown that miR-146a–mediated IRAK1 suppression in IECs is essential for immune TLR pathway tolerance to gut bacteria in neonatal intestine.^16^^,^^24^ We therefore examined whether decreased miR-146a and TLR sensitivity noted in *Sigirr*^*Tg*^ mice correlates with persistence of IRAK1 protein in mouse IECs. IRAK1 (a miR-146a target) protein expression levels were increased in IECs as shown in IEC lysates on DOL-11 in heterozygote and homozygous *Sigirr*^*Tg*^ mice and by immunofluorescence (Figure 6*E* and *F*). STAT3^Ser727^ phosphorylation in IECs was decreased in *Sigirr*^*Tg*^ mice compared with littermate controls, while total STAT3 expression levels were comparable (Figure 6*E*). These data indicate that *Sigirr*^*Tg*^ mice have decreased IEC expression of anti-inflammatory microRNAs in association with decreased STAT3 phosphorylation and increased IRAK1 expression.

### IRAK1–STAT3 Mediated microRNA Expression in Mouse Intestinal Epithelium

**Figure 7:**
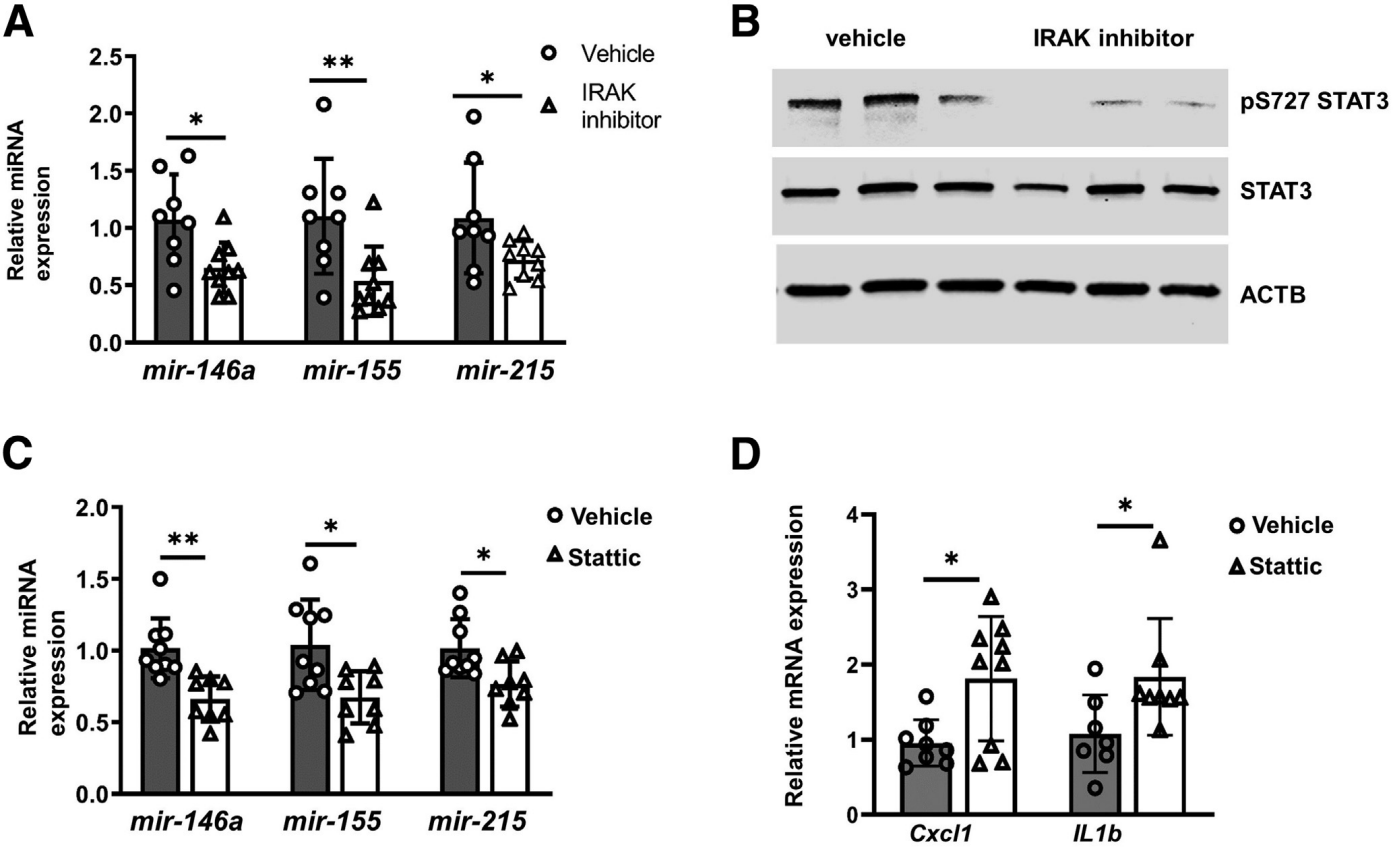
**Decreased intestinal expression of microRNAs and increased inflammation in wild-type neonatal mice treated with IRAK and STAT3 inhibitor.** (*A*) microRNA expression in mouse IECs from vehicle or IRAK inhibitor–treated mice were quantified by real-time PCR. DOL-6 wild-type mice were orally administered IRAK inhibitor at 20 mg/kg of body weight once per day for 3 days, then killed for IEC isolation and analysis. (*B*) Mouse IEC lysates were analyzed by immunoblotting with the indicated antibodies. (*C* and *D*) Real-time PCR analysis of expression of microRNA and proinflammatory cytokines in IECs isolated from wild-type mice with 3 days intraperitoneal injection of STATTIC at 2.5 mg/kg of body weight daily from DOL-7. ∗*P* < .05, ∗∗*P* < .01. ACTB, actin beta.

Because microRNA expression is regulated by IRAK1–STAT3 signaling in human IECs in vitro, we examined conservation of this signaling pathway in developing intestine using wild-type mice. DOL-6 wild-type pups were orally administered IRAK1 inhibitor at 20 mg/kg or vehicle once a day for 3 days. Stat3 Ser727 phosphorylation in IECs treated with IRAK inhibitor was decreased significantly compared with vehicle control. miR-146a, miR-155, and mir-215 levels were suppressed owing to IRAK1 inhibition (Figure 7*A*). Reduced STAT3 Ser727 phosphorylation was observed in IRAK1-inhibitor–treated IECs, indicating that IRAK1 phosphorylated STAT3 and regulated microRNAs expression in vivo (Figure 7*B*). Similarly, inhibition of STAT3 with the inhibitor decreased miRNA expression and induced proinflammatory cytokine expression in mouse ileum (Figure 7*C* and *D*) epithelium. These results also were observed in ileal IECs from neonatal mice treated with the STAT3 inhibitor, Stattic. Together, these data suggest that the SIGIRR–IRAK1–Stat3 axis regulates anti-inflammatory microRNA expression in the neonatal intestinal epithelium.

## Discussion

The neonatal intestinal mucosa has to rapidly adapt to a new environment containing commensal and pathogenic bacteria after birth. As a major negative regulator, SIGIRR canonically inhibits TLR and IL1 receptor (IL1R)-mediated inflammation through competitively binding MYD88 and inhibiting TIR homology domain interactions between TLR/IL1R and MYD88 immediately after ligand stimulation.^25^^,^^26^ Whether SIGIRR regulates TLR signaling through post-translational inhibition of key TLR signaling pathway effectors and the mechanisms involved remain unknown. Our results identified a novel inhibitory SIGIRR signaling cascade via STAT3–miRNA–146a that suppresses IRAK1, a known mediator of postnatal intestinal tolerance. Using in vitro approaches in HIECs and a novel mouse that encodes a mutation we discovered in human NEC, we show that SIGIRR regulates expression of anti-inflammatory microRNAs through IRAK1-dependent, STAT3-mediated transcriptional activation. SIGIRR-mediated miR-146a and miR-155 expression in the neonatal gut inhibits IRAK1 expression, and loss of the SIGIRR TIR domain seen with the SIGIRR p.P174X mutation results in TLR hyper-responsiveness and spontaneous inflammation in neonatal intestine. These data provide new insights into the acquirement of postnatal intestinal TLR tolerance.

MiRNAs are key regulators of inflammatory responses and immune regulation.^27^^,^^28^ MiR-146a is one of the first anti-inflammatory miRNAs identified to be involved in innate immune responses.^29^ After birth, miR-146a expression is greatly induced and maintained at a high level during the neonatal stage, and miR-146a–mediated IRAK1 suppression is critical for acquirement of innate immune tolerance to colonizing bacterial in the neonatal intestine.^16^ However, the mechanism by which miR-146a expression is induced and maintained in the neonatal intestinal epithelium still is unclear. Our data show decreased miR-146a in SIGIRR-deficient primary human IECs and in *SIGIRR* mutant mouse neonatal intestine epithelium, indicating that SIGIRR plays a major role in the regulation of miR-146a expression in the neonatal intestinal epithelium. Similar to miR-146a, miR-155 is another mitigator of inflammatory response that targets MYD88, the mediator of TLR and IL1R signaling.^28^ miR-215 is emerging as an anti-inflammatory microRNA.^19^ MiR-155 and mir-215 are greatly decreased in the intestines of NEC patients,^17^^,^^18^ and we also observed that these 2 microRNAs are decreased in HIECs with the p.Y168X mutation, and in the small intestine IECs of mice encoding a truncating SIGIRR mutation (p.P174X) that phenocopies the human mutation. These data suggest that miR-155 and mir-215 may be potential targets for the prevention and treatment of hyperactive TLR-induced intestinal injury in neonates. Although we focused on mir-146a, mir-155, and mir-215, whether other microRNAs, such as let-7 and mir-21,^14^ which inhibit TLR signaling, were altered by SIGIRR loss of function needs to be investigated in the future.

STAT3 activation through IL6 or IL22 in IECs is a major inhibitor of the intestinal inflammation through several mechanisms including IL10 secretion.^30^^,^^31^ However, the role of STAT3-dependent miRNA in repressing TLR signaling in the neonatal intestine, and its induction by SIGIRR, is poorly understood. Here, we identified that the SIGIRR TIR domain activates STAT3 through phosphorylation and mediates STAT3-dependent anti-inflammatory microRNA expression in neonatal intestinal epithelium. STAT3 is known to regulate miR-146a expression in hepatocellular carcinoma cells and miR-155 in T-helper 17 cells.^21^^,^^22^ Given the role of miR-146a and miR-155 in innate immune response,^28^ we propose that STAT3-regulated microRNA expression in IECs is important for containing intestinal TLR signaling in response to colonizing bacteria. This aids neonatal intestinal immune tolerance, which is lost in mice genocopying a NEC-related human *SIGIRR* mutation. Our results show that SIGIRR-dependent STAT3 activation results in its binding to the promoter of anti-inflammatory microRNAs and inducing their expression in intestine epithelial cells. Dysregulated STAT3 function reduced expression of anti-inflammatory microRNAs both in HIECs and mouse intestine, resulting in failure of SIGIRR to suppress flagellin-induced inflammation in HIECs and increased proinflammatory cytokine expression in the mouse intestine. More importantly, administration of miR-146a mimic rescued loss of function related to the p.Y168X mutant in relation to TLR5-induced inflammation. These results show a new mechanism by which SIGIRR inhibits TLR canonical signaling through STAT3-mediated miRNA expression, not only through the conventional MYD88–NF-κB pathway. The embryonic lethality of *Stat3*^*-/-*^ mice precluded us from probing its direct role in neonatal intestinal miRNA expression, although use of an inhibitor supports our conclusions. Other members of the STAT family are reported to be involved in regulation of intestinal inflammation.^32^^,^^33^ Whether other STAT family members are regulated by SIGIRR is an interesting topic for future studies.

We discovered that IRAK1 is the SIGIRR downstream kinase, which phosphorylates STAT3 at Ser727 in intestinal epithelial cells. Our data are consistent with previous work that has shown that IRAK1 can phosphorylate STAT3 at Ser727 in splenocytes.^34^ The functional impact of IRAK-mediated STAT3 Ser727 phosphorylation is demonstrated by our data showing decreased miRNA expression in HIECs and mouse IECs after IRAK inhibition despite the presence of SIGIRR. During TLR-/IL1R-mediated innate immune response, TLR or IL1R bind to MYD88 through their cytoplasmic TIR domains, forming a Myddosome complex along with IRAK4 and IRAK1, resulting in activation of downstream signaling.^35^, ^36^, ^37^ Previously, Lotz et al^24^ showed that postnatal acquirement of neonatal intestinal tolerance in mice is related to decreases in IRAK1 protein, although IRAK1 mRNA levels remained stable, suggesting post-translational modification. Subsequently, Chassin et al^16^ showed that miR-146a suppressed IRAK1 translation, although their results suggested that low-level IRAK1 is critical for maintaining miR-146a expression in the presence of intraepithelial endotoxin. Our results showing that SIGIRR represses IRAK1 protein and low levels of IRAK1 expression is adequate for SIGIRR-dependent, STAT3-mediated miR-146a expression is consistent with Chassin et al.^16^ Our co-immunoprecipitation data suggest that SIGIRR can bind MYD88 at baseline via TIR domain assembly and may constitutively activate IRAK1 to phosphorylate STAT3 for maintaining microRNA expression even without ligand stimulation. Impairing SIGIRR expression or function by siRNA or TIR domain truncation mutant, respectively, decreased STAT3 phosphorylation both in vitro and in vivo, indicating that constitutive SIGIRR–MYD88 complex can activate IRAK1 kinase activity and contribute to anti-inflammatory microRNA expression, critical for silencing TLR signaling.

Postnatal acquirement of neonatal intestinal tolerance to bacteria through tamponade of TLR4 signaling is important for the prevention of NEC. Several mechanisms including induction of miR-146a–mediated IRAK1 suppression and induction of inhibitor of nuclear factor kappa B (IκB), which sequesters NF-κB in the cytoplasm, are important.^16^^,^^24^^,^^38^ How miR-146a is induced in the neonatal intestine, facilitating IRAK1 suppression and TLR4 insensitivity, is unknown. To better understand SIGIRR regulation of neonatal intestinal adaptation, we used CRISPR-Cas9 genome editing to generate *Sigirr*^*Tg*^ mice with a p.P174X mutation, which truncates the TIR domain, mimicking the p.Y168X mutation identified in human NEC.^13^ Combining loss-of-function/gain-of-function of experiments in human fetal IECs with studies in *Sigirr*^*Tg*^ mice, we identified a novel mechanism (SIGIRR–STAT3–miR-146a) in the neonatal intestine by which SIGIRR represses IRAK1 and facilitates neonatal immune tolerance. Loss of this mechanism in *Sigirr*^*Tg*^ mice resulted in a dose-dependent increase in neonatal intestinal inflammation at baseline. We believe our results provide new insights into how mutations in *SIGIRR*, a potential locus for NEC susceptibility in premature infants, create a milieu of intestinal TLR hypersensitivity that might portend neonatal gut injury phenotypes such as NEC. Future studies will examine how mutations in *SIGIRR* and other potential NEC susceptibility genes interact with the microbiome to prime TLR sensitivity and program NEC vulnerability.^3^^,^^39^

## Materials and Methods

### Ethical Approval

Care of mice before and during experimental procedures was conducted in accordance with the policies at the University of Missouri–Kansas City Laboratory Animal Resource Center and the National Institutes of Health Guidelines for the Care and Use of Laboratory Animals. Protocols had prior approval from the University of Missouri–Kansas City Institutional Animal Care and Use Committee. Investigators understand the ethical principles and the work described here complies with the animal ethics checklist published in *Cellular and Molecular Gastroenterology and Hepatology*.

### Generation of New Mice

*Sigirr* p.P174X mutant mice were generated by pronuclear injection of a CRISPR plasmid expressing Cas9 and single-guide RNA into 1-cell C57BL/6 embryos. Founder animals were genotyped and confirmed by Sanger sequencing to identify a founder animal harboring a 32-bp deletion, which generates a premature stop at amino acid 174. This mutant founder was back-crossed to the parental strain to establish a breeding colony. All subsequent litters were genotyped using the following primers: forward: 5′-CTCTTTAACTGGCCCTGCTG-3′; reverse: 5′-CAGGGAACAGAGTAGGGACC-3′.

### Mouse Inhibitor Studies

For inhibitor treatments, DOL-6 C57BL/6J (B6) pups were orally administered the IRAK inhibitor (cat. no. 5665; Tocris, Minneapolis, MN) at 20 mg/kg for 3 days. For STAT3 inhibition, DOL-6 pups were injected intraperitoneally with STAT3 inhibitor, Stattic (cat. no. sc-202818; Santa Cruz Biotechnology, Santa Cruz, CA) at 2.5 mg/kg for 3 days.

### Cell Culture

HIEC-6 (CRL-3266; American Type Culture Collection, Manassas, VA) cells were grown in OptiMEM 1 Reduced Serum Medium supplemented with 4% fetal bovine serum (FBS), 20 mmol/L HEPES, 10 mmol/L GlutaMAX(ThermoFisher, Waltham, MA), and 10 ng/mL epidermal growth factor in an atmosphere containing 5% CO_2_. Transfection was performed using Lipofectamine 3000 (ThermoFisher) according to the manufacturer’s protocol. Human embryonic kidney HEK293T cells were cultured in Dulbecco's modified Eagle medium containing 10% FBS and antibiotic–antimycotic (ThermoFisher). HIECs transduced by lentiviral particle expressing Stat3 shRNA or control shRNA were passaged 2 times to eliminate the effect of lentivirus. HIECs were transfected with pcDNA3 vectors(Invitrogen, Waltham, MA) carrying wild-type SIGIRR or p168X mutant^3^ using Lipofectamine 3000 according to the manufacturer’s protocol. Three days after transfection, HIECs were lysed for mRNA and protein expression analysis. For RNA interference, the specific siRNA duplexes targeting human SIGIRR (sc-61547), fluorescein-conjugated control siRNA (sc-36869), transfection reagent (sc-29528), and reduced-serum transfection medium (sc-36868) were purchased from Santa Cruz Biotechnology. When the cell confluence reached 60%–80%, the cells were washed with 1× phosphate-buffered saline (PBS) and transiently transfected with transfection complexes prepared according to the manufacturer’s instructions. At 48 or 72 hours after transfection, silencing of SIGIRR at mRNA and protein levels was checked by real-time PCR and Western blot, respectively.

### Isolation of Mouse Small Intestine Epithelial Cells

Mouse small intestine epithelial cells were isolated as previously reported.^40^ Briefly, 5 cm terminal ileum was flushed with cold PBS, opened longitudinally, and cut into 1-cm pieces, followed by incubation in 5 mL Hank’s balanced salt solution (Ca2+ Mg2+ free) with 2% FBS, 5 mmol/L EDTA, and 1 mmol/L dithiothreitol in a 37°C shaker for 30 minutes. Cells were collected by passing supernatant through a 100-μm cell strainer, and spinning at 300 × *g* for 5 minutes. Isolated cells were confirmed positive for epithelial cells markers E-cadherin and epithelial cell adhesion molecule (EpCAM) by immunofluorescence.

### ChIP

The ChIP experiment was performed as previously described.^41^ Briefly, the HIECs were fixed with 1% formaldehyde for 10 minutes. The Pierce Magnetic ChIP Kit and ChIP grade Stat3 antibody (#4904; Cell Signaling, Danvers, MA) were used according to the manufacturer’s instructions. ChIP products were analyzed by quantitative real-time PCR. The sequences of primers were designed to target Stat3 binding sites at microRNA promoters according to the prediction of JASPAR 2018 (http://jaspar.genereg.net/) and as listed: miR-155 forward: 5’-AAAGTTCCAGATCAAGGTCCTG-3’; miR-155 reverse: 5’-AGAGACACCAGAATTCCCTTTC-3’; miR-146a forward: 5’-TCCAAGCATCCACTTTCCTG-3’; miR-146a reverse: 5’-GTACATTATCCCGTTCCAGTCAG-3’; mir-215 forward: 5’-TGGAACGCAGACCTTACAAG-3’; and mir-215 reverse: 5’-AGTGGGATATAACAGGGATTTCAG-3’.

### Luciferase Assay

The 2-kb promoter of miR-146a was PCR-amplified from the human complementary DNA (cDNA) library and cloned into pGL4.10 (Promega, Madison, WI). To gain high transfection efficiency, HEK293T cells were used for luciferase assays. HEK293T cells were co-transfected with pGL4.1–miR-146a–Luc and pcDNA3–Stat3, Stat3 S727A pRc/CMV (Addgene, Watertown, MA), or pCDNA3-SIGIRR. The thymidine kinase promoter–Renilla luciferase reporter plasmid served as an internal control. At 48–72 hours after transfection, luciferase and Renilla signals were measured using a Dual-Luciferase Reporter Assay Kit (Promega) by the CLARIO star multiplate reader (BMG Labtech, Ortenberg, Germany).

### Quantitative Real-Time PCR

Total RNA was isolated using TRIzol (ThermoFisher). cDNA was synthesized using a high-capacity cDNA reverse-transcription kit (Bio-Rad, Hercules, CA). For the quantification of gene amplification, real-time PCR was performed using a ViiA7 or Quantstudio 3 (Applied Biosystems, Waltham, MA). The sequences of gene-specific primers are available upon request. 18S ribosomal RNA or glyceraldehyde-3-phosphate dehydrogenase were used as endogenous normalization control.

### Western Blot Analysis

The Western blot analysis was performed as described previously. In brief, cells were washed once with ice-cold PBS and suspended in RIPA lysis buffer (Pierce, Waltham, MA), added with a cocktail of protease inhibitors. Mouse tissues in RIPA buffer containing protease and phosphatase inhibitors were homogenized by bullet blender (Midwest Scientific, St. Louis, MO). The concentration of the crude protein was measured with a bicinchoninic acid Protein Assay Kit (ThermoFisher Scientific). Equal amounts of cell lysates were loaded and separated by sodium dodecyl sulfate–polyacrylamide gel electrophoresis, and gels were blotted to polyvinylidene difluoride membranes. Membranes with blotted proteins were incubated with primary antibodies, washed, and incubated with peroxidase-conjugated secondary antibodies. Reactive proteins were shown with an enhanced chemiluminescence detection system and visualized on the imaging system iBright FL1000 (ThermoFisher). Densitometry was performed using ImageJ software (National Institutes of Health, Bethesda, MD), and changes were normalized to actin beta (ACTB) or the corresponding nonphosphorylated antibody.

### Immunostaining

Paraffin sections of intestine were cut in 4-um thickness. Sections were deparaffinized with xylene and graded series of alcohol. Antigen retrieval was performed using citrate buffer for 20 minutes at 95°C. After several rinses with PBS, sections were blocked with Power Block Universal Blocking Reagent for 1 hour and then stained overnight at 4°C with primary anti-SIGIRR (rabbit, 1:30; MyBioSource, San Diego, CA) and anti-IRAK1 (rabbit, 1:100; Abcam, Cambridge, UK ). After washing with PBS, slides were stained with secondary antibody conjugated with Alexa488 (1:100; Invitrogen, Waltham, MA) for 1 hour at room temperature.

For frozen sections, freshly isolated intestine tissue was fixed in ice-cold 4% paraformaldehyde for 6 hours, rinsed several times with ice-cold PBS, and then left overnight at 4°C in 30% sucrose. Tissue was embedded in optimal cutting temperature (OCT) compound and cryosectioned with 10-um thickness. Sections were washed several times with PBS and blocked with Power Block (Biogenex Laboratories, Fremont, CA) for 1 hour and then stained with primary anti-Stat3–phospo-S727 (rabbit, 1:100; Abcam) overnight at 4°C. After washing with PBS, secondary antibody conjugated with Alexa488 (1:100; Invitrogen) was added and kept for 1 hour at room temperature.

In both paraffin and frozen sections, after treating with secondary antibodies the slides were washed several times with PBS, counterstained with 4′6′-diamidino-2-phenylindole, and mounted with FLUORO-GEL (1798510; Electron Microscopy Science, Hatfield, PA). All primary and secondary antibodies were diluted in Antibody Diluent Solution (Invitrogen). Images were acquired using ×25/oil objective with a Zeiss Inverted LSM 510 meta laser scanning confocal microscope(Oberkochen, Germany). Red–green–blue images were assembled using ImageJ software.

### Protein Structure Construction

The sequence of SIGIRR (Uniprot Q6IA17; www.uniprot.org/) was parsed into extracellular and intracellular parts followed by modeling each with I-TASSER (https://zhanggroup.org/I-TASSER/),^42^ joining the two with the transmembrane helix, energy minimizing, and embedding within a lipid membrane using the YASARA tools (http://www.yasara.org/).^43^ The SIGIRR and MYD88 dimer model was generated by merging the Toll-interleukin receptor domain homology models from protein data bank (PDB) files 1T3G. SIGIRR P115R 2-dimensional images were generated by Phyre2.

### Data Analysis

Data are presented as means ± SD or as the median with interquartile range. *P* < .05 was considered significant. For cell culture experiments, data are from a minimum of 3 independent experiments with adequate technical replicates used for quantification. All animal data were obtained in littermate controls. For animal experiments, a minimum of 4 animals were used for each experimental group. RNA quantification and PCR results had 2–3 technical replicates. Statistical analysis was performed using GraphPad Prism 9.0 (San Diego, CA). For all data, we initially examined whether distribution of data was Gaussian using the D'Agostino–Pearson omnibus normality test. Comparisons between 2 groups were made by a 1-sample, 2-tailed Student *t* test for parametric or nonparametric data. Comparisons between 3 or more groups were analyzed by 1-way analysis of variance and post hoc Tukey tests for multiple comparisons.
